# Brain glucose metabolism during hypoglycemia in type 1 diabetes: insights from functional and metabolic neuroimaging studies

**DOI:** 10.1007/s00018-015-2079-8

**Published:** 2015-10-31

**Authors:** Hanne M. M. Rooijackers, Evita C. Wiegers, Cees J. Tack, Marinette van der Graaf, Bastiaan E. de Galan

**Affiliations:** 1 Department of Internal Medicine 463, Radboud University Medical Center, PO Box 9101, 6500 HB Nijmegen, The Netherlands; 2 Department of Radiology and Nuclear Medicine, Radboud University Medical Center, Nijmegen, The Netherlands; 3 Department of Pediatrics, Radboud University Medical Center, Nijmegen, The Netherlands

**Keywords:** Brain metabolism, Hypoglycemia, Type 1 diabetes mellitus, Impaired awareness of hypoglycemia, Neuroimaging, Cerebral blood flow

## Abstract

Hypoglycemia is the most frequent complication of insulin therapy in patients with type 1 diabetes. Since the brain is reliant on circulating glucose as its main source of energy, hypoglycemia poses a threat for normal brain function. Paradoxically, although hypoglycemia commonly induces immediate decline in cognitive function, long-lasting changes in brain structure and cognitive function are uncommon in patients with type 1 diabetes. In fact, recurrent hypoglycemia initiates a process of habituation that suppresses hormonal responses to and impairs awareness of subsequent hypoglycemia, which has been attributed to adaptations in the brain. These observations sparked great scientific interest into the brain’s handling of glucose during (recurrent) hypoglycemia. Various neuroimaging techniques have been employed to study brain (glucose) metabolism, including PET, fMRI, MRS and ASL. This review discusses what is currently known about cerebral metabolism during hypoglycemia, and how findings obtained by functional and metabolic neuroimaging techniques contributed to this knowledge.

## Introduction

The brain is one of the most metabolically active organs in the body and it consumes energy disproportionate to its size. In humans, the brain represents only about 2 % of total body weight, yet it accounts for approximately 20 % of the body’s oxygen use and 25 % of the body’s use of glucose. Glucose is the primary fuel for the adult brain. In young adults, the ‘resting’ brain consumes approximately 110 g of glucose per day, i.e. 5.5 mg glucose per 100 g of brain tissue per minute [1]. Since the brain’s energy stores are small, normal brain function depends on a continuous supply of glucose from the bloodstream. Under normal conditions, the human body takes great effort and is very efficient in avoiding hypoglycemia in almost all circumstances to maintain sufficient glucose delivery to the brain.

Type 1 diabetes mellitus is an autoimmune-mediated disease, characterized by destruction of most, if not all, of the insulin-producing capacity of pancreatic beta-cells. As a consequence, supplemental insulin treatment is required to maintain glucose control and decrease the risk of complications resulting from hyperglycemia [2]. Unfortunately, therapeutic insulin is still poor at mimicking the pharmacology of endogenous insulin. As a consequence, people with type 1 diabetes—in particular those aiming for strict glycemic control—are at continuous risk of hypoglycemia, the average frequency of which has been estimated at two non-severe, symptomatic episodes per week [3–5] and one severe, potentially hazardous event, per year [4, 6, 7]. Although there is substantial variation in both the rate and the severity of hypoglycemia, both between and within persons [4, 8], this estimation means that the brains of people with type 1 diabetes are exposed to many thousands of hypoglycemic episodes over a lifetime of diabetes.

Studying brain metabolism during hypoglycemia may reveal the potential harmful effects of (recurrent) hypoglycemia on the brain and may increase our understanding of metabolic adaptations that might underlie impairments in the defenses against hypoglycemia [9]. Modern neuroimaging techniques have enabled the study of cerebral metabolism in vivo in a relatively non-invasive manner. This review will focus on the effect of hypoglycemia on brain (glucose) metabolism, with a particular emphasis on recent findings from functional and metabolic neuroimaging studies. Basic mechanisms of brain energy metabolism and neuroimaging techniques will be discussed briefly.

## Glucose counterregulation

In healthy, non-diabetic humans, hypoglycemia is unlikely to ever occur due to a hierarchically coordinated system that integrates insulin secretion and counterregulatory hormone and symptom responses [10, 11]. When glucose levels in the low-physiological range (e.g. late post-absorptive or fasting state) tend to fall, insulin secretion is suppressed to such an extent that true hypoglycemia can almost always be prevented. When insulin is given to experimentally induce hypoglycemia in people without diabetes, glucose levels at or below ~3.8 mmol/L will induce a glucagon response, the secretion of which by pancreatic alpha-cells is probably controlled by the neighboring beta-cells [10, 11]. Such a glucose level also stimulates the secretion of adrenaline, whereas slightly lower levels are needed to elicit autonomic warning symptoms, such as sweating, palpitations, trembling and feeling hungry [10, 11]. These symptoms are aimed at initiating a behavioral response (i.e. ingesting carbohydrates). Further falls in plasma glucose values result in neuroglycopenic symptoms, which range from mild cognitive impairment, such as difficulty in concentrating, to overt confusion and even coma or seizures in its most severe form [10, 11].

In patients with type 1 diabetes, hypoglycemia typically results from the interplay between therapeutic peripheral hyperinsulinemia and impaired defenses against falling plasma glucose levels [10]. These impairments first include the inability to decrease insulin and to increase glucagon in response to hypoglycemia. The latter is not a structural defect, but specific for hypoglycemia and probably secondary to loss of control by non-functioning beta-cells [12]. In patients with longer diabetes duration and more frequent exposure to hypoglycemia, adrenaline responses to hypoglycemia become attenuated, in part due to a shift of these responses to lower glucose values [13]. The defective adrenaline responses are associated with, although not necessarily the cause of, similar defects in the emergence of autonomic symptom responses [14, 15]. Disappearance of these symptoms interferes with the ability to timely and accurately perceive, interpret and respond to falling plasma glucose levels. This inability is known as the clinical syndrome of impaired awareness of hypoglycemia and increases the risk of particularly severe hypoglycemia, defined as those events requiring assistance from another person [16], by a factor of six or more [17, 18]. Both the attenuated adrenaline response and impaired awareness of hypoglycemia are usually the result of (recurrent) antecedent hypoglycemia rather than of autonomic neuropathy, for which the term ‘hypoglycemia-associated autonomic failure’ (HAAF) has been introduced [19]. HAAF can be effectively treated by several weeks to months of scrupulous avoidance of hypoglycemia [15, 20, 21], although it appears that the symptomatic component responds earlier and better than the hormonal component [15]. The underlying mechanism(s) explaining the attenuating effect of prior hypoglycemia on responses to subsequent events have not been fully elucidated. However, there is agreement that alterations in the brain play a pivotal role.

## Morbidity associated with hypoglycemia

The glucose level at which cognitive function declines is subject to substantial variation; in some people cognitive dysfunction already occurs at plasma glucose levels between 3.0 and 4.0 mmol/L, whereas others continue to function well at levels below 2.5 mmol/L [8, 22]. Almost all domains of cognitive function are potentially at risk during acute hypoglycemia, with complex tasks (e.g. car driving) being affected earlier than simple tasks [23, 24]. Prolonged and/or profoundly severe hypoglycemia may eventually cause neuronal death [25, 26]. The cerebral cortex and hippocampus are the most vulnerable regions in the brain to be injured by severe hypoglycemia, while the brain stem and cerebellum are most resistant [27]. However, although persistent vegetative states or brain death resulting from hypoglycemia have been described [28–30], most patients with type 1 diabetes recover uneventfully from even severe hypoglycemia complicated by seizures or coma, especially when they are young and in good clinical condition. In addition, evidence for an association between multiple episodes of severe hypoglycemia and long-term cognitive decline in people with type 1 diabetes is lacking [31, 32]. Finally, although 4–10 % of all deaths in patients with type 1 diabetes have been attributed to hypoglycemia, most of these deaths are thought to be either accidental (e.g. in traffic) or cardiovascular (e.g. arrhythmia) rather than the direct consequence of brain death [33, 34].

It should be noted that both the developing brain of young children with type 1 diabetes [35, 36] and the brain of the elderly, in particular in patients with type 2 diabetes [37, 38], seem more susceptible to harm from hypoglycemia. Children with type 1 diabetes performed worse on cognitive tests when they had a history of severe hypoglycemia below the age of 5 years, compared to patients without such a history and non-diabetic controls [35]. In prospective cohorts of people with type 2 diabetes, a history of severe hypoglycemia has been associated with cognitive decline and frank dementia [38], as well as with greater risks of cardiovascular events and death [39, 40]. On the cellular level, there are now indications that glucose deprivation may accelerate apoptosis of neurons, which could underlie neuronal cell death and predispose for cognitive decline [41]. It has also been speculated that the acute, physiological changes in hematological and hemodynamic parameters induced by hypoglycemia may be particularly damaging when the vasculature has already been injured [42, 43], possibly explaining the discrepancy between type 1 and type 2 diabetes [31, 44]. Another factor explaining this discrepancy may lie in the concept of hypoglycemic preconditioning. Rodents exposed to recurrent hypoglycemic events of moderate severity were less likely to develop neuronal damage or cognitive impairments or die during subsequent severe hypoglycemia than age-matched littermates who were not pre-exposed to hypoglycemia [45, 46]. These data may help to explain recent observations that patients with type 1 diabetes and impaired awareness of hypoglycemia, as a reflection of recurrent hypoglycemia, appeared not at greater risk of dying than patients with intact awareness [47].

## The role of the brain in glucose counterregulation

The brain is not just at the receiving end of hypoglycemia, but it plays an important role in both the detection of hypoglycemia and in the subsequent initiation and coordination of counterregulatory responses to restore euglycemia, as described above. This system maintains glucose homeostasis through a classic sensory-motor integrative pathway in which a decrease in plasma glucose levels is detected by an extended network of glucose sensing neurons located within the brain and the periphery [48]. Specialized glucose-sensing cells are located in the hepatic portal/mesenteric vein, gut, carotid body and oral cavity. In the brain, glucose-sensing neurons are found at a number of locations, but particularly in the ventromedial nucleus of the hypothalamus (VMH) and areas that originate from the hindbrain. Integrative networks receive projections from these sensing neurons and subsequently assimilate their input with signals from other brain regions, such as information about circadian rhythm and arousal state. This information is relayed to motor neurons, which generate an output that drives the counterregulatory response and subsequently restores plasma glucose levels. Conversely, glucose sensing may influence other neural processes that have no role in glucose counterregulatory function [49].

Although the VMH is only one of a number of regions involved in the detection of hypoglycemia, it is thought to be the most important. The VMH serves as the central relay station for signals from many other regions and plays a crucial role in the coordination of the counterregulatory responses to falling glucose levels. Animal studies have shown that both destruction of the VMH and local perfusion of the VMH with glucose, disrupt counterregulatory hormone responses to systemic hypoglycemia [50, 51]. Conversely, local glucopenia in the VMH stimulates these responses in the absence of hypoglycemia [52]. In analogy, glucose counterregulation was also found defective in a patient with lesions from sarcoidosis in the hypothalamus, presumably due to destruction of the glucose-sensing neurons in the VMH [53].

The mechanism of glucose sensing by the brain, in particular in the VMH, has not been fully clarified. Two main types of glucose-sensing neurons have been identified: glucose-excited neurons, whose activity increases as glucose levels rise, and glucose-inhibited neurons, which become more active as glucose levels fall and less active when they rise [54]. These neurons ‘sense’ a fall in glucose probably as a result of alterations in ATP/ADP and AMP/ATP ratios, respectively, following a reduction in glucose metabolism. This could explain why fuelling the VMH with an alternative source of energy, such as lactate, suppresses glucose counterregulation [55, 56]. The subsequent intracellular actions that may ultimately lead to a counterregulatory response probably involve activation of AMP-activated protein kinase, formation of nitric oxide and release of glutamate in glucose-inhibited neurons. Other potential mediators involved in these responses include (a decrease in) gamma-aminobutyric acid (GABA) release from glucose-excited neurons, noradrenaline, serotonin and corticotrophin-releasing hormone [57]. For further reading on this subject, we refer to recent reviews by McCrimmon [54] and Chan and Sherwin [57].

## Cerebral glucose delivery, uptake and metabolism

Glucose is transported across the blood–brain barrier into extracellular fluid (ECF) by facilitated diffusion, mediated via glucose transporter protein 1 (GLUT1) [58]. The predominant transporters involved in subsequent glucose uptake from the ECF in neurons and in astrocytes are GLUT3 and GLUT1, respectively [59], both insulin-independent glucose transporters. Once intracellular, glucose is phosphorylated by hexokinase as the initial step of glucose metabolism. The glucose-6-phosphate (Glc-6-P) thus produced can enter several metabolic pathways in the brain [60].

In 1945, Kety and Schmidt developed the first method to quantitatively assess brain glucose uptake in humans in vivo and to derive data on its subsequent metabolism [61]. This highly invasive technique required the use of arterial and internal jugular vein catheterizations to determine arteriovenous concentration differences for glucose, which together with measurement of global cerebral blood flow (CBF) were then used to calculate the global cerebral metabolic rate of glucose [61–64]. In humans, the Kety–Schmidt method was used to show that brain glucose uptake falls during hypoglycemia and that this coincides with the appearance of counterregulatory hormone responses and autonomic warning symptoms [65, 66]. However, whether these data can be used to reliably assess brain glucose metabolism is a matter of debate. Indeed, the calculations rely solely on the disappearance rate of glucose from the circulation. Therefore, this technique cannot discriminate between specific metabolic steps and ignores the potential contribution of other metabolites. Moreover, the highly invasive nature of the Kety–Schmidt technique is a considerable limitation for research in humans.

**Fig. 1:**
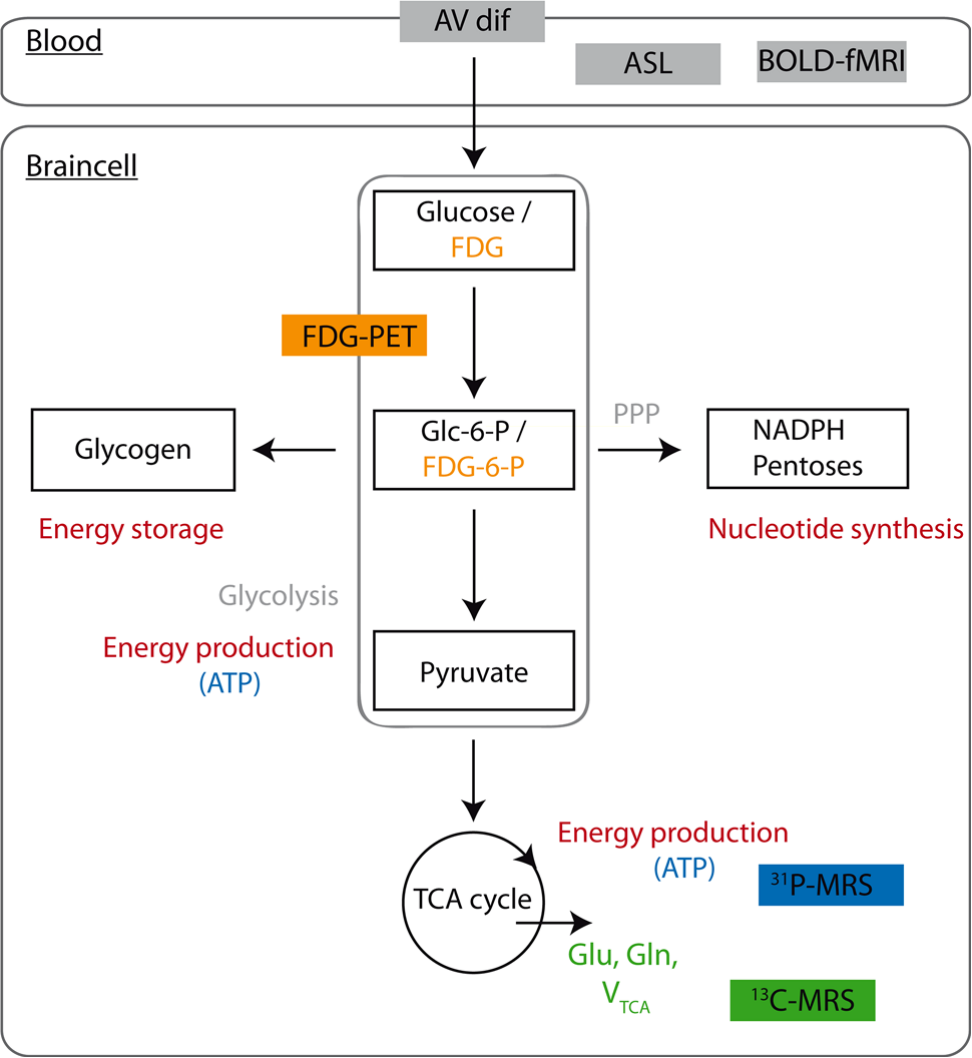
A simplified illustration of the multiple metabolic pathways of glucose in the brain and the metabolic signals used in different neuroimaging techniques. The initial step of glucose metabolism is phosphorylation of glucose to glucose-6-phosphate (Glc-6-P) by hexokinase. Glc-6-P can enter several metabolic pathways in the brain. It can be metabolized to produce energy via glycolysis or the TCA cycle. Glycolytic and TCA cycle intermediates are also used for the synthesis of amino acids and neurotransmitters. In addition, Glc-6-P is a precursor for glycogen. Lastly, metabolism of Glc-6-P via the pentose phosphate pathway (PPP) provides pentose for nucleotide synthesis and NADPH, required for reductive reactions, such as lipid synthesis and for protection against oxidative stress. Arteriovenous concentration differences (AV dif) can be used to estimate global cerebral metabolic rate from the disappearance of metabolites from the circulation. PET (depicted in *orange*) uses radiolabeled glucose analogues (such as FDG), which are trapped early in metabolism (for example fluorodeoxyglucose-6-phosphate/FDG-6-P), to estimate rates of glucose uptake and metabolism. ^31^P MRS (depicted in *blue*) provides information about ATP production and thus brain energy metabolism. ^13^C-MRS (depicted in *green*) is useful for estimating TCA cycle fluxes and CMR_glc_, derived from ^13^C label incorporation into specific metabolites (Glu, Gln). Both ASL and BOLD fMRI provide estimates of CBF

The past 40 years have shown rapid advances in modern metabolic and functional neuroimaging techniques to study brain (glucose) metabolism vis-à-vis CBF during hypoglycemia, including positron emission tomography (PET), functional magnetic resonance imaging (fMRI) and magnetic resonance spectroscopy (MRS). It is important to note that the distribution of CBF and the cerebral metabolic rate of glucose (CMR_glc_) are closely linked to local brain activity. Brain activation causes proportionate increases in both local CBF and CMR_glc_. These processes are being referred to as neurovascular coupling or neurometabolic coupling, respectively, and hypothesized to be mediated by neurotransmitter release and vasoactive metabolic products [67]. Many functional neuroimaging techniques, including fMRI, rely on neurovascular coupling. The principles of the various imaging techniques will be briefly discussed (Fig. 1).

### PET

Positron emission tomography can be used to measure emissions from a variety of radioactively labeled tracers in the brain to quantify CBF, glucose uptake and phosphorylation, oxygen consumption and brain receptors for major neurotransmitters, depending on the type of radiotracer used [68]. ^15^O-labeled water PET, for example, has been commonly applied to quantify regional CBF [69, 70]. For the study of brain glucose metabolism, [^18^F]fluoro-2-deoxy-d-glucose (FDG) is the most widely used tracer. FDG is taken up by the brain in a similar manner as glucose, but unlike native glucose, once phosphorylated (FDG-6-P), it cannot be metabolized further, resulting in accumulation of the tracer in the cell. Under steadystate conditions, in which total influx of metabolites into a pathway equals the outflow, the rate of tracer accumulation in the brain can be used to estimate global and regional rates of glucose transport and metabolism [58]. PET has been particularly valuable in studying the effect of hypoglycemia on CBF, brain glucose uptake and cerebral metabolic rate in humans with and without type 1 diabetes [71–74]. However, this technique cannot be used to study glucose metabolism downstream of its conversion to glucose-6-phosphate [75]. Also, animal studies suggest that the lumped constant, a correction factor that relates the metabolic rate of FDG to that of native glucose [76], may increase during hypoglycemia [77, 78]. The tracer [^11^C]3-*O*-methyl-d-glucose (3-OMG) may provide more robust information about cerebral glucose uptake at varying glucose concentrations, as it is not phosphorylated [79], but its relative short half-life time (~20 min) and complex preparation limits the use of this compound in a clinical setting [80].

### fMRI

Functional magnetic resonance imaging is primarily used to study regional neuronal activation (patterns) by the detection of changes in oxygen demand by the brain [81], based on the concept of neurovascular coupling described above. Blood oxygenation level dependent (BOLD) contrast is one of the primary contrast mechanisms for fMRI, which exploits the differences in magnetic properties between deoxygenated and oxygenated hemoglobin [82]. Regional brain activation increases local oxygen demands, but because the consequent increase in CBF exceeds these demands, the balance between deoxygenated and oxygenated hemoglobin changes towards the latter. This change in hemoglobin oxygenation can be probed and detected, so that a brain map of regions with increased or decreased activation can be constructed [81]. fMRI has been especially useful in detecting brain activation patterns in response to specific cognitive tasks or visual stimulation. Hypoglycemia has been reported to reduce regional BOLD activation in response to these tasks, but less so in patients with type 1 diabetes [83] than in non-diabetic subjects [84, 85]. These reductions in BOLD responses are commonly attributed to decreased neuronal activity, yet the potential impact of hypoglycemia on (global or regional) CBF, neurovascular coupling or oxidative metabolism, remains to be determined.

### ASL

Arterial spin labeling (ASL) is an MRI method that provides non-invasive quantification of global and regional CBF. ASL does not require an exogenous contrast agent, but uses magnetically labeled arterial blood water as a diffusible tracer. Arterial blood water is first labeled magnetically using radiofrequency (RF) pulses. Subsequently, this labeled arterial blood flows into the brain where it exchanges with tissue water, after which an image is taken. The experiment is then repeated without labeling the arterial blood to create a control image. The signal difference between control and labeled images reflects local CBF [86, 87]. While the signal-to-noise (SNR) ratio in BOLD fMRI is higher, ASL measures brain perfusion more directly, enables quantification of CBF, and is suitable for studying variations in CBF over a longer period of time due to stable noise characteristics [88]. ASL thus allows the detection of changes in CBF during hypoglycemia and is, in contrast to fMRI, less dependent on other metabolic parameters, such as oxygenation or glucose concentrations that might change during hypoglycemia. A high magnetic field (e.g. 3 T) is usually recommended to improve SNR when performing ASL.

### MRS

Magnetic resonance spectroscopy is a non-invasive technique, closely related to MRI. Both techniques make use of the spin properties of certain nuclei when brought into a magnetic field. For MRI, the proton nucleus is used to construct a highly detailed anatomical image based on the different water concentrations in various tissues. For MRS, these spin properties are used to determine the concentration of specific metabolites in the tissue examined. These concentrations are derived from the peaks in a spectrum [89]. MRS is feasible on any nucleus possessing a magnetic moment, but is most frequently performed on the high natural abundant and MR sensitive proton nucleus (^1^H), providing steady-state information on concentrations of proton-containing brain metabolites at a single time point [90]. However, because water contains most of the proton nuclei, the water signal needs to be suppressed to allow reliable measurements of metabolite concentrations. As a consequence, the sensitivity of MRS is manifold lower than that of MRI, even at high magnetic fields. Nevertheless, since nearly all metabolites contain protons, ^1^H-MRS is a powerful technique to identify and quantify a large number of metabolites relevant for glucose metabolism (e.g. lactate, glutamate, glutamine) at in vivo concentrations typically above 0.5 mM.

**Fig. 2:**
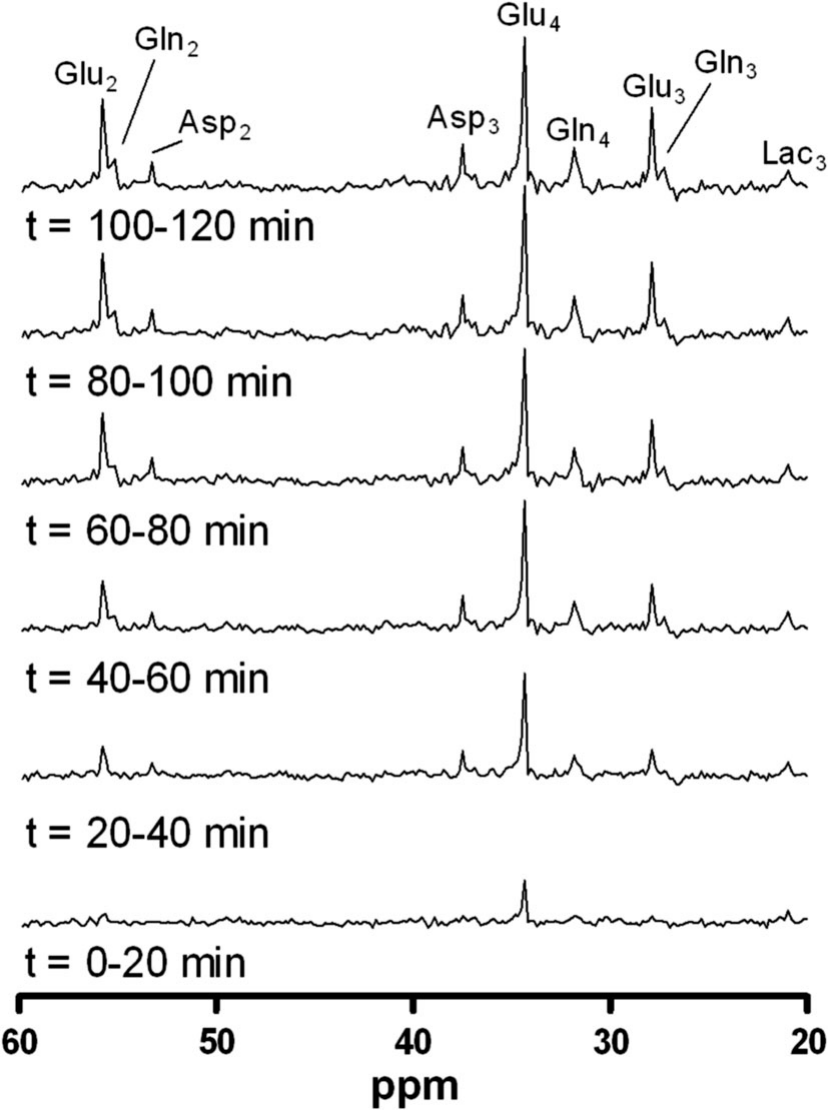
Time series of ^13^C-MR spectra, acquired from a ~125 mL voxel, placed in the occipital cortex. Spectra are averaged over 20 min, after administration of [1-^13^C]glucose during a hypoglycemic clamp in one healthy subject. Once the infused [1-^13^C]glucose is taken up by the brain and incorporated into various glucose metabolites, an increase in signal over time is observed. Numbers indicate the position of the ^13^C label, as explained in more detail in Fig. 3. *Asp* aspartate, *Gln* glutamine, *Glu* glutamate, *Lac* lactate (from Ref. [143], with permission from Elsevier)

The use of carbon-13 (^13^C) in MRS is specifically relevant for the study of brain glucose metabolism. Carbon exists in the human body in two isotopes, of which carbon-12 (^12^C) is dominant with a natural abundance of 98.9 %. ^12^C does not possess a net nuclear spin and consequently cannot be detected by MRS. In contrast, ^13^C does possess a magnetic moment, but has a very low natural abundance of 1.1 %. However, the intravenous infusion of ^13^C-enriched substrates, such as [1-^13^C]glucose, [3-^13^C]lactate or [3-^13^C]acetate, offers the possibility to study fluxes of these substrates in the brain through important metabolic pathways (Fig. 2).

**Fig. 3:**
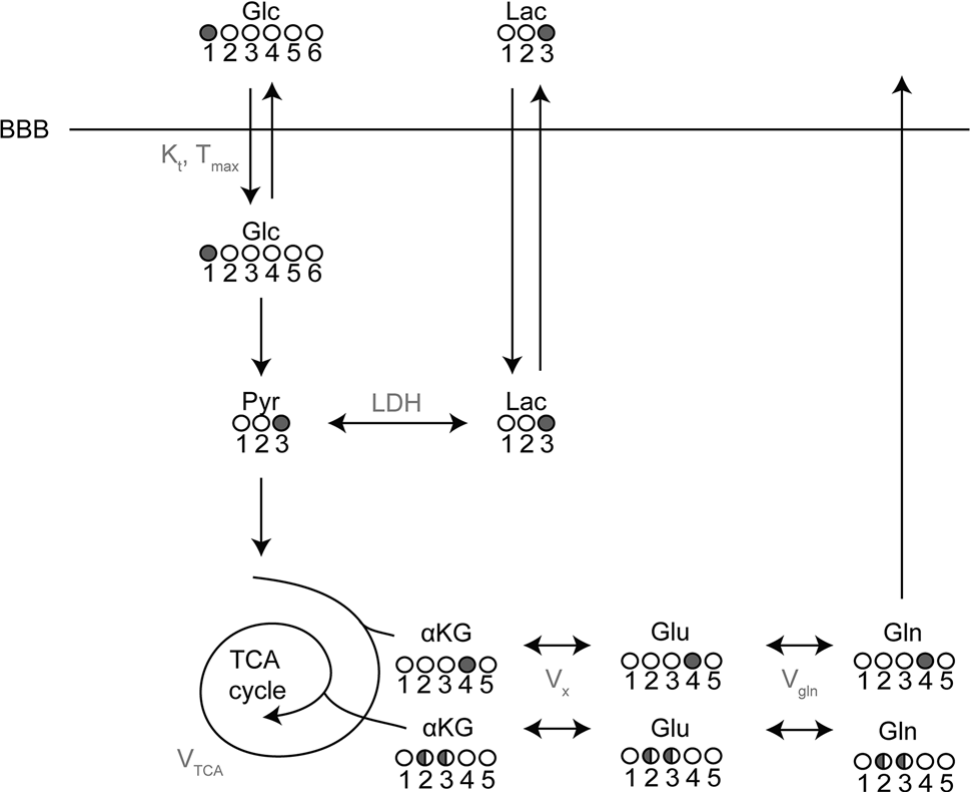
One-compartment metabolic model describing the incorporation of ^13^C label from (infused) [1-^13^C]glucose into the TCA cycle and its metabolites. When taken up by the brain, the ^13^C-label is first incorporated into the C3 position of pyruvate and subsequently into the C3 position of lactate. Once the ^13^C-label continues through the TCA cycle, it is incorporated into the C4 position of αKG, glutamate and glutamine. In the second turn of the cycle, the label is equally distributed over the C2 and C3 positions of these metabolites. The TCA cycle flux can be estimated using a metabolic model where the time courses of the uptake of the ^13^C-label in glutamate and glutamine in the different carbon positions, measured with ^13^C-MRS, are used as input. *Filled circles* represent the carbon position that is labeled with ^13^C, *white circles* represent unlabeled carbons. *αKG* α-ketoglutarate, *BBB* blood–brain-barrier, *Glc* glucose, *Gln* glutamine, *Glu* glutamate, *Lac* lactate, *LDH* lactate dehydrogenase, *Pyr* pyruvate, *V*_*gln*_ exchange rate between glutamate and glutamine, *V*_*TCA*_ TCA cycle rate, *V*_*x*_ exchange rate between α-ketoglutarate and glutamate

For the study of brain glucose metabolism, it is important to note that [1-^13^C]glucose is taken up and metabolized by the brain similar to native (i.e. unlabeled) glucose. Following transport across the blood–brain barrier and phosphorylation by hexokinase, glucose is the main substrate for the production of energy (by formation of ATP) via glycolysis and the tricarboxylic acid (TCA) cycle [82]. As such, ^13^C-MRS allows the fate of the ^13^C-labeled glucose to be followed as it flows into glycolysis. The ^13^C-label is transferred from glucose on the C-1 position to pyruvate on the C-3 position during glycolysis and, subsequently, passes through all metabolites of the TCA cycle. In this process, the ^13^C-label is incorporated into the MRS-detectable metabolites glutamate, glutamine and aspartate, all at specific carbon positions. Because these positions change during the second time the isotope flows in the TCA cycle (Fig. 3), the time-course of ^13^C-label incorporation into these metabolites can be used as input for a metabolic model to calculate the TCA cycle flux and CMR_glc_ [91]. However, although the fates of individual carbon atoms can be tracked in the TCA cycle, cerebral ^13^C-MRS provides no information about the loss of label in diffusible metabolites, such as glutamine and lactate which may exchange between brain and blood plasma [82, 92].

^13^C-MRS has been proven to be a valuable imaging technique to study brain (glucose) metabolism via specific pathways in humans in vivo, under various conditions, including hyperglycemia [93] and hypoglycemia [94]. In addition, ^13^C labeled compounds other than glucose, such as ^13^C-acetate and ^13^C-lactate, can be used to provide a more complete picture of the very complex metabolic processes in the brain. Indeed, ^13^C-acetate, which is metabolized almost exclusively in astroglia [95], has been used to distinguish astroglial and neuronal metabolism more directly, and to study transport and metabolism of non-glucose fuels during hypoglycemia [96, 97].

Phosphorus-31 (^31^P) is another naturally abundant nucleus with a relatively high sensitivity for MRS. ^31^P-MRS of the brain can be used to detect metabolites that play a key role in brain energy metabolism and provides information on flux through the creatine kinase reaction (e.g. ATP, phosphocreatine, inorganic phosphate), intracellular pH and magnesium concentrations [98]. Thus far, this technique has been seldom used to study brain metabolism during hypoglycemia [99].

## Cerebral nutrient transport capacity and hypoglycemia

### Glucose uptake

**Fig. 4:**
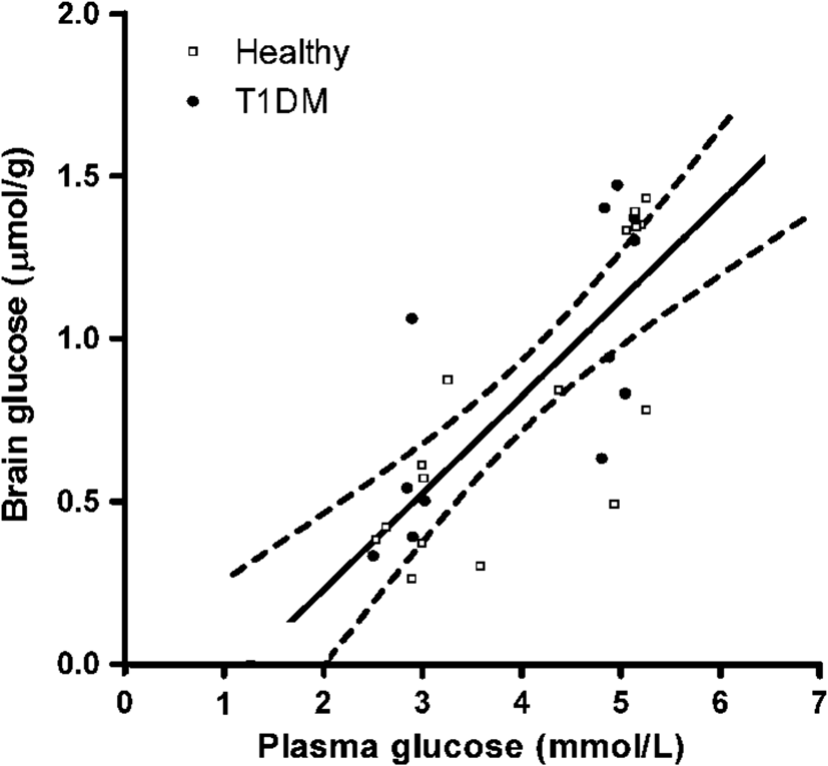
Linear relationship between plasma and brain glucose levels under normo- and hypoglycemic conditions in healthy subjects (*open squares*) and patients with type 1 diabetes (*closed circles*). Brain glucose levels were measured with ^13^C-MRS. The plasma versus brain glucose relation was fitted with linear regression analysis to determine reversible Michaelis–Menten kinetics to show the best fit of the data with 95 % confidence intervals. *R* ^2^ = 0.59, *P* < 0.001. Assuming continuation of this linear relationship between plasma and brain glucose levels, brain glucose approaches zero at a plasma glucose level of approximately 1.2 mmol/L (from Ref. [104], with permission from the American Diabetes Association)

As mentioned above, glucose uptake into the brain occurs through facilitated transport independent of insulin. As a consequence, there is a linear relationship between plasma glucose concentrations and brain glucose content over a range of pla**s**ma glucose values up to ~30 mmol/L [100–103]. This linear relationship also extends well into the hypoglycemic range, although data below plasma levels of ~2.5 mmol/L are missing in humans (Fig. 4) [104]. To explain HAAF, it has been hypothesized that chronic or repeated hypoglycemia increases glucose transport capacity over the blood–brain barrier to compensate for the fall in glucose availability to the brain during subsequent hypoglycemia. Indeed, several animal studies have shown that days to weeks of chronic hypoglycemia cause upregulation of brain glucose transporters, including both GLUT-3 on neuronal membranes [105, 106], and GLUT-1 on the vascular endothelium at the blood–brain barrier [107, 108]. In accordance, Boyle and co-workers applied the Kety and Schmidt technique to show preservation of brain glucose transport rather than a fall during hypoglycemia in healthy volunteers after prior exposure to hypoglycemia, whereas it fell when such exposure had not taken place [65]. The investigators went on to report similar findings of preserved glucose transport in patients with type 1 diabetes and near-normal glycosylated hemoglobin (HbA_1c_), possibly reflecting high hypoglycemic burden, as they also had reduced awareness of hypoglycemia [66].

In mice and in rats, very low plasma glucose values, typically well below 2.0 mmol/L, have been found to proportionally increase brain glucose uptake as a function of increased cerebral perfusion [102, 109]. Various neuroimaging studies investigating glucose transport over the blood–brain barrier in humans have produced conflicting results. A ^1^H-MRS study performed under hyperglycemic conditions showed greater brain glucose concentrations in patients with type 1 diabetes and impaired awareness of hypoglycemia than in people without diabetes [110]. However, a similar study found no evidence of altered brain glucose transport in healthy volunteers subjected to antecedent repeated hypoglycemia, despite clearly attenuated hormone responses to hypoglycemia [111]. In accordance, global blood-to-brain glucose transport, as measured with [1-^11^C]-glucose PET, remained unaltered in healthy volunteers after exposure to 24 h of moderate hypoglycemia, albeit interspaced with transient glucose normalizations during meals [112]. Finally, a 3-OMG-PET study also showed no differences in global brain glucose transport during hypoglycemia between patients with normal and those with impaired awareness of hypoglycemia [72].

### Monocarboxylic acid (MCA) uptake

Although glucose is its principal source of energy, the brain may resort to alternative non-glucose fuel substrates under glucopenic conditions. These alternative substrates include foremost ketones, and lactate, which enter the TCA cycle after conversion to pyruvate or acetyl coenzyme A, and can be metabolized in a similar way as glucose to sustain brain metabolism, and spare glucose.

Ketones such as beta-hydroxybutyrate and acetoacetate are synthesized in the liver from fatty acids during prolonged fasting, starvation and severe carbohydrate restriction. Under such conditions, up to 60 % of brain energy requirements may be derived from ketone metabolism [113], whereas ketogenic diets can more or less restore brain energy metabolism and prevent epileptic seizures in patients with GLUT1 deficiency who are unable to transport glucose into the brain [114, 115]. However, because insulin suppresses the production of ketones, the brain is usually unable to use this source of energy during insulin-induced hypoglycemia [116]. PET studies with the use of both ketone and glucose tracers may help to unravel the complex interaction between the metabolism of ketones and glucose by the brain under different circumstances, including hypoglycemia [113].

In recent years, it has gradually been recognized that lactate plays an important role in the energy metabolism in the brain, particularly during hypoglycemia since both hypoglycemia and insulin increase plasma levels of lactate, at least in healthy subjects [94, 117, 118]. Under basal, euglycemic, conditions, the contribution of systemic lactate to cerebral energy metabolism is approximately 8–10 %. However, the proportional contribution of lactate has been reported to increase during strenuous exercise, when plasma lactate levels rise substantially [119, 120]. The role of lactate in specific areas of the brain includes its involvement in or interference with hypoglycemia detection in the VMH as stated above. Lactate has also been found to be a crucially monitored variable in the detection of energy imbalance in the caudal hindbrain [121]. The importance of lactate for the brain was first highlighted when Pellerin and Magistretti published their astrocyte-neuron lactate shuttle (ANLS) hypothesis. This hypothesis posits that glucose is taken up by and metabolized in astrocytes to form lactate, after which lactate is exported to neighboring neurons where it is oxidized, especially during activation [122]. This concept, which bears analogy to the cell–cell lactate shuttle, through which skeletal muscle can transport a non-glucose energy source to other organs [123], thus suggests that astrocytes play the primary role in brain glucose metabolism. Simpson et al. later came to a different conclusion and developed a model that basically adopts the opposite view, in which neurons are the principal site of glucose uptake and metabolism, and the chief exporter of lactate. This hypothesis was therefore termed the neuron-astrocyte lactate shuttle (NALS) [59] and fuelled a heavy debate [124, 125]. The debate on the direction of the lactate shuttle is ongoing with studies identifying the neuron as the principal locus of glucose uptake [126], and other studies indicating that neurons rather than astrocytes are the primary sites for oxidation of exogenous lactate [119, 127].

Monocarboxylic acid transporters (MCTs) facilitate the uptake of lactate as well as that of acetate and ketone bodies into the brain, the expression of which may increase following sustained hyperketonemia or recurrent hypoglycemia. A recent study in rats demonstrated a twofold increase in the expression of MCTs 1 and 2 in the cerebral cortex after the induction of diabetes by streptozotocin. After 8 weeks of frequent, prolonged endurance training and concomitant exposure to hypoglycemia after and between exercise sessions, the expression of both transporters increased even further [128]. Such greater transport capacity may explain recent observations in which recurrent exposure to hypoglycemia increased the uptake of ^13^C-labeled lactate into the rat brain under hypoglycemic conditions [129]. During hypoglycemia, the uptake of both acetate and lactate into the human brain, as measured by ^13^C-MRS during infusion of ^13^C-labeled acetate or lactate, respectively, was found to be considerably greater in patients with well-controlled type 1 diabetes than in healthy controls [97, 130].

### Transport and uptake of other substrates

Oral intake of amino acids has been reported to enhance the glucagon response to hypoglycemia and to improve some aspects of cognitive function during hypoglycemia in non-diabetic and diabetic subjects [131, 132]. Amino acids might also serve as a non-glucose substrate that could be used by the brain as an alternative fuel and to sustain cognitive function during hypoglycemia. Early studies showing utilization of amino acids by the rat brain during prolonged hypoglycemia and of amino acids contributing to glycogen synthesis in brain cell cultures supported this theory [133, 134]. However, data obtained in humans using arteriovenous concentration differences found no evidence that greater availability of amino acids increased its net brain uptake during hypoglycemia [135] or was able to offset energy deficit due to reduced glucose supply [136].

A few studies have investigated whether the human brain can use lipid substrates to support cerebral metabolism and brain function during hypoglycemia. Fatty acids can readily cross the blood–brain barrier to be oxidized by the brain, as demonstrated by a ^13^C-MRS study in rats [137]. In healthy humans, elevated plasma levels of non-esterified fatty acids and glycerol were found to reduce hormonal and symptom responses to hypoglycemia, but could not protect against the fall in cognitive function [138]. Conversely, in a more recent study, ingestion of medium-chain triglycerides maintained cognitive function during hypoglycemia without affecting adrenergic or symptomatic responses to hypoglycemia in intensively treated subjects with type 1 diabetes [139]. It should be acknowledged, however, that the inferences made with respect to the uptake of lipid substrates in the brain were indirect and that no neuroimaging studies have been performed that evaluated the effects of these substances on cerebral metabolism more directly.

## Brain metabolism during hypoglycemia

### Glucose metabolism

Both PET and MRS have been used to investigate the effect of hypoglycemia on brain glucose metabolism. PET has been particularly useful in detecting regional differences in tracer accumulation in the brain, both during hypoglycemia [71], and after restoration to euglycemia [74]. However, rather than focusing on glucose uptake or metabolism, the close link with neuronal activation is then exploited to use the data as input factors for mapping regional brain activity. Thus, the observation that CMR_glc_ relatively increased during hypoglycemia in patients with type 1 diabetes and normal awareness of hypoglycemia, and relatively fell in patients with impaired awareness of hypoglycemia, was interpreted as an increase in brain activation and absence of such a response [72]. When this increased activation would occur in brain areas involved in the perception of and the generation of responses to hypoglycemia, the lack of increased activation could then underlie loss of hypoglycemic awareness [72, 140]. Support for this hypothesis came from another FDG-PET study [141] and a subsequent analysis of these data [73], as tracer uptake in areas that engage appetite control and food-seeking behavior was reduced in patients with impaired compared to patients with intact awareness of hypoglycemia.

As outlined above, ^13^C-MRS in combination with infusion of ^13^C-labeled glucose has the unique property that it enables the investigation of cerebral glucose metabolism in humans in vivo. Since the SNR is relatively low, most studies employing this technique used large doses of isotopically enriched glucose at high enrichment percentages. Measurements have consequently generally been performed under hyperglycemic conditions with glucose levels up to 17 mmol/L and plasma C-13 enrichment values exceeding 60 %. Under such conditions, Henry et al. [93] reported no differences in the TCA cycle rate between patients with type 1 diabetes with impaired awareness of hypoglycemia and healthy controls. More recently, an improved sensitivity of the ^13^C-MRS method [142] in combination with an optimized ^13^C-glucose infusion protocol enabled us to study glucose metabolism in the human brain during hypoglycemia at lower enrichment values [143]. With this optimized technique, no differences were observed in cerebral glucose metabolism between hypoglycemia and euglycemia, neither in healthy controls [94], nor in patients with type 1 diabetes [144]. Under hypoglycemic conditions, however, the TCA cycle rate was approximately 45 % higher in patients than in healthy subjects, and inversely related to HbA_1c_. Appreciating a low HbA_1c_ as a proxy for a high hypoglycemic burden, these data suggested a role for prior hypoglycemic exposure in the higher TCA cycle rate in patients with type 1 diabetes. Differences in brain glucose levels did not explain the preservation of brain metabolism and the higher TCA cycle rate in the patients, which suggested influx of a non-glucose carbohydrate source [104]. In an animal study by Herzog et al. [129], brain glucose transport capacity during hypoglycemia became rate limiting for TCA cycle activity in control animals, but not in rats exposed to antecedent recurrent hypoglycemia. Explanations for the discrepancy between the human and rodent data include the different species and the fact that the hypoglycemic condition was more profound in the animals. Indeed, studies in mice suggest that intracellular brain glucose concentrations approach depletion at plasma glucose values between 2 and 3 mmol/L [109].

### Glycogen metabolism

The brain is able to store glycogen and to use this compound when plasma glucose levels are low, although its capacity to do so is very limited compared to other tissues such as skeletal muscle and the liver. It was long assumed that this presence of glycogen was restricted to astrocytes. However, a recent study showed that neurons contain a low but measurable amount of glycogen, the use of which was found to protect against hypoxic stress, at least in neuronal cell cultures and animal models [145]. Both in rodents [146] and in humans [147], it was shown that brain glycogen was used during hypoglycemia, and that its stores were replenished above baseline levels after restoration of euglycemia, a phenomenon termed glycogen supercompensation. It has been speculated that this expanded source of glucose within the brain could contribute to the development of impaired awareness of hypoglycemia by fuelling the brain or at least those areas involved in glucose-sensing during subsequent hypoglycemia [146]. However, prior exposure to recurrent hypoglycemia neither facilitated nor impaired access to glucose from glycogen in the rat brain during subsequent hypoglycemia [148]. Additionally, brain glycogen content, as measured by ^13^C-MRS in conjunction with ^13^C-glucose administration, was lower rather than higher in patients with type 1 diabetes and hypoglycemia unawareness [149].

### Glutamate metabolism

Glutamate is the major excitatory neurotransmitter in the brain, but has many other metabolic fates, including the formation of glutamine, GABA and glutathione [150]. In addition, a new concept has been introduced by Sonnewald [151], who proposed that glutamate degradation in astrocytes contributes to most of the lactate that is released from the brain under resting conditions, offering a novel explanation for the concept of aerobic glycolysis in the resting state [151, 152]. Lastly, glutamate can be oxidized for the production of energy [150]. To this end, glutamate production in the brain is tightly coupled to TCA cycle activity [153]. Using ^1^H-MRS, Bischof et al. reported that hypoglycemia reduced the cerebral glutamate to creatine ratio in healthy controls, but not in patients with type 1 diabetes [154]. Similar results were reported by a more recent ^1^H-MRS study, were hypoglycemia reduced brain glutamate levels in healthy controls and in patients with type 1 diabetes with normal hypoglycemic awareness, but not in patients with impaired awareness of hypoglycemia [155]. The authors concluded that the preservation of brain glutamate during hypoglycemia in the latter group reflected a metabolic adaptation that eliminated the need to oxidize glutamate. They speculated that this adaptation could be augmented transport of glucose or of alternative fuels to the brain.

### Metabolism of monocarboxylic acids

As discussed earlier, MRS studies using ^13^C-labelled acetate and lactate have clearly suggested that the capacity to transport MCAs over the blood–brain barrier during hypoglycemia is increased in patients with well-controlled type 1 diabetes. Indeed, a study during which ^13^C-acetate was infused under hypoglycemic conditions showed more than twofold higher brain acetate concentrations in subjects with type 1 diabetes compared to healthy controls. This greater acetate availability translated into a fraction of oxidative metabolism that resulted from acetate to be similarly increased [97]. In accordance, the relative contribution of acetate to brain metabolism in rats exposed to recurrent antecedent hypoglycemia was also increased during next-day hypoglycemia, indicating that brain substrate preferences may change rapidly from glucose to alternative substrates if needed [156]. To delineate whether this effect was a function of diabetes, prior hypoglycemia or both, the investigators repeated their ^13^C-acetate study in patients with type 1 diabetes with normal or impaired awareness of hypoglycemia and in healthy controls. They found that absolute rates of acetate metabolism during hypoglycemia were only higher in the patients with impaired awareness of hypoglycemic, suggesting that changes in acetate metabolism are the consequence of prior exposure to hypoglycemia rather than of diabetes per se [96].

Lactate uses the same MCT as acetate to cross the blood–brain barrier. Since plasma levels of lactate are approximately tenfold higher than those of acetate, and hypoglycemia stimulates the production of lactate [117], it seems plausible that lactate is the more likely substrate for brain metabolism when glucose levels are low. Studies dating back to the 1990s have shown that exogenous administration of lactate attenuates counterregulatory responses *to* and preserves cognitive function *during* hypoglycemia, presumably because lactate is used as an alternative source of energy by the brain [157–160]. In agreement, brain lactate concentrations during hypoglycemia, derived from the cerebral uptake of ^13^C-labelled lactate, were several fold higher in patients with type 1 diabetes with a history of frequent hypoglycemic episodes than in non-diabetic subjects [130] and in rats exposed to recurrent hypoglycemia versus those not exposed [129]. Surprisingly, the authors found no indication of greater lactate oxidation, as reflected by unchanged ^13^C fractional enrichments of brain glutamate and glutamine [130]. Data from the rodent study, in which lower glucose levels were achieved than in the human study, suggested that prior hypoglycemic exposure increased both the uptake and the oxidation of glucose by the brain, despite the higher lactate levels [129]. However, when the animal brain was stimulated during hypoglycemia, animals exposed to recurrent hypoglycemia had a partial loss of their functional cortical response, which was only normalized after the administration of lactate. This suggests that the higher capacity for lactate transport only becomes critical when the brain is activated during (deep) hypoglycemia.

## Cerebral blood flow and hypoglycemia

There is uncertainty as to whether hypoglycemia affects global CBF and in what direction. Previous research in both patients with type 1 diabetes and healthy controls has reported either no change in global CBF during hypoglycemia [65, 66, 136], a modest increase [161–163], or even a slight decrease [71]. Differences in the plasma glucose levels achieved during hypoglycemia and, more importantly, in imaging techniques probably explain many of the discrepancies. Studies that investigated the effect of hypoglycemia on regional relative changes in CBF seem to have produced more consistent data. Both in healthy controls [161] and in patients with type 1 diabetes [164], hypoglycemia was found to increase blood flow to the frontal lobes. This relative redistribution of regional CBF was already observed under euglycemic conditions in patients with type 1 diabetes, and was more pronounced in patients who had experienced frequent hypoglycemia [165]. Since the frontal lobes are among the most vulnerable brain areas to suffer structural damage, this may be an adaptive response to prevent such damage by maintaining fuel supply during subsequent hypoglycemia.

Hypoglycemia has also been found to increase CBF in the thalamus [71, 166, 167] and hypothalamus [168, 169]. Mild or moderate hypoglycemia caused a rise in CBF in the hypothalamus in healthy non-diabetic subjects, as assessed by fMRI [168] or ASL [169], which preceded the rise in counterregulatory hormone responses seen during hypoglycemia [169]. Interestingly, Mangia et al. found blunting of this increase in thalamic perfusion during hypoglycemia in patients with type 1 diabetes with hypoglycemia unawareness, and a correlation between thalamic perfusion and the adrenaline response to hypoglycemia [167]. In contrast, recurrent hypoglycemia enhanced, rather than decreased, thalamic perfusion during subsequent hypoglycemia in healthy controls [170], so that the role of this brain region in the adaptation to hypoglycemia remains uncertain.

## Discussion

The scientific field of metabolic and functional neuroimaging techniques for the brain has tremendously progressed over the past couple of decades. The application of these techniques to hypoglycemia research has considerably advanced our understanding of the brain’s responses to hypoglycemia. As plasma glucose falls below levels that can be reversed by responses at the level of pancreatic islets, i.e. suppression of insulin release and stimulation of that of glucagon, the brain’s sensing abilities are activated to allow timely detection of hypoglycemia. Data from functional and metabolic neuroimaging techniques now suggest that such moderate hypoglycemia neither affects the perfusion to nor the uptake of glucose into the brain, at least not globally, unless much deeper levels of glucose are achieved [102, 109]. In accordance, cerebral glucose *metabolism* appears largely maintained during moderate hypoglycemia [94, 129, 144]. However, on the regional level, moderate hypoglycemia causes redistribution of CBF to various brain areas involved in the detection of hypoglycemia, particularly the (hypo)thalamus [71, 166–169], where enhanced neuronal activation stimulates glucose uptake and metabolism. Such enhanced neuronal activation has also been found to occur in brain areas involved in appetitive motivational networks [73], thus linking the detection of hypoglycemia to a behavioral response.

Modern neuroimaging studies have revealed that recurrent hypoglycemia, which typically affects people with type 1 diabetes and underlies the clinical syndrome of impaired awareness of hypoglycemia, may initiate cerebral adaptations at many different levels. First, there is interference with the accurate detection of hypoglycemia, probably occurring at the level of the VMH. Brain areas that control appetite and induce fear and anxiety may not become activated during hypoglycemia. Whether locally increased glucose uptake in or reduced neuronal activation of the hypothalamic area (or both) form the underlying mechanism remains to be revealed. Importantly, it should be acknowledged that neurovascular coupling may be altered as a consequence of diabetes per se [171], chronic hyperglycemia [172] or microangiopathy [173], thus limiting the interpretation of studies relying on this concept. There is conflicting evidence as to whether recurrent hypoglycemia can stimulate brain glucose uptake during hypoglycemia [65, 66], although most studies employing neuroimaging techniques found no evidence for this suggestion [72, 111, 112]. Nevertheless, patients with type 1 diabetes, particularly those with impaired awareness of hypoglycemia [155], seem better able in maintaining brain (glucose) metabolism during hypoglycemia than healthy controls [94, 144, 154, 155], probably as a consequence of prior hypoglycemia [144]. Since profound hypoglycemia will eventually cause brain glucose metabolism to deteriorate [129], such an adaptation may shift the threshold for deterioration of the metabolic rate to lower plasma glucose levels.

Several mechanisms have been proposed that could explain the discrepancy between hypoglycemia-induced preservation of cerebral glucose metabolism and the fall in glucose availability during hypoglycemia. It seems likely that influx of a non-glucose energy substrate plays a role. Recent (neuroimaging) studies found little evidence to support enhanced blood to brain transport of amino acids [135, 136] or lipid substrates transport [138, 139] and ketones are unlikely candidates because its production is suppressed by insulin. Also, the lower brain glycogen content in patients with type 1 diabetes with impaired awareness of hypoglycemia compared to controls [149] argues strongly against the glycogen supercompensation hypothesis. Several arguments suggest a major role for lactate in preserving brain glucose metabolism during hypoglycemia. These include: (1) lactate can be used by the brain and may even be preferred over glucose under non-hypoglycemic conditions [174, 175]; (2) the capacity for lactate transport over the blood–brain barrier is increased in patients with impaired awareness of hypoglycemia and in rats after exposure to hypoglycemia [97, 129]; (3) the use of lactate by glucose-sensing neurons in the VMH may interfere with hypoglycemia sensing [54, 56, 57]. However, there are data that suggest that lactate is not used as major energy source for the brain during moderate hypoglycemia, despite greater availability [129, 130]. Also, it is not yet known whether brain uptake or metabolism of endogenously produced lactate is increased during hypoglycemia in patients with type 1 diabetes and impaired awareness of hypoglycemia. Finally, it has been suggested that lactate may serve as a metabolic regulator or intercellular signaling molecule rather than a fuel, modulating brain glucose metabolism, oxygen delivery and CBF [176, 177]. Mechanisms by which lactate might exert this effect [178] include modulation of prostaglandin action (and thus CBF) [177, 179], adjustment of the NADH/NAD^+^ redox ratio [123], and the regulation of neuronal cAMP formation via the lactate receptor G-protein-coupled receptor 81 (GPR81) [180].

## Conclusion

Hypoglycemia is the principal barrier for achieving optimal, let alone normal, glycemic control for indefinite periods of time in patients with type 1 diabetes and advanced insulin-requiring type 2 diabetes [181]. Recurrent hypoglycemia forms the basis of HAAF and the clinical syndrome of impaired awareness of hypoglycemia by attenuating physiological defenses against subsequent hypoglycemia, consequently increasing the risk for severe hypoglycemia. Paradoxically, the mechanism(s) underlying these glucose counterregulatory impairments may be related to, or even caused by, processes that are seemingly aimed at protecting the brain against harm from severe hypoglycemia. The progress in metabolic and functional neuroimaging techniques has revealed that recurrent hypoglycemia causes cerebral adaptations to occur on many different levels. These adaptations include those in the regional delivery (blood flow) and transport of glucose to the brain, the handling of glucose by the brain and that of non-glucose alternative fuels, as well as activation or de-activation of brain areas involved in behavioral responses. It remains to be elucidated whether, and if so under which circumstances and in which brain areas, the brain uses non-glucose alternative sources of energy, particularly lactate, and whether this contributes to the emergence of impaired awareness of hypoglycemia. Such information is needed first to foster personalized decision-making with respect to glycemic targets, but should eventually lead to treatments that eliminate hypoglycemia from the lives of people with type 1 diabetes without compromising glucose control.

## Abbreviations

ASL: Arterial spin labeling
ANLS: Astrocyte-neuron lactate shuttle
BOLD: Blood oxygenation level dependent
CBF: Cerebral blood flow
CMR_glc_: Cerebral metabolic rate of glucose
CMRO_2_: Cerebral oxygen metabolic rate
ECF: Extracellular fluid
FDG: ^18^F-Fluoro-2-deoxy-d-glucose
fMRI: Functional magnetic resonance imaging
GABA: Gamma-aminobutyric acid
Glc-6-P: Glucose-6-phosphate
GLUT: Glucose transporter protein
GPR81: G-protein-coupled receptor 81
HAAF: Hypoglycemia-associated autonomic failure
HbA_1c_: Glycosylated hemoglobin
MCA: Monocarboxylic acid
MCTs: Monocarboxylic acid transporters
MRS: Magnetic resonance spectroscopy
NALS: Neuron-astrocyte lactate shuttle
PET: Positron emission tomography
RF: Radiofrequency
SNR: Signal to noise ratio
TCA: Tricarboxylic acid
VMH: Ventromedial hypothalamus
3-OMG: ^11^C-3-*O*-methyl-d-glucose
^13^C: Carbon-13
^31^P: Phosphorus-31
